# Supplementary material for: KSHV reprograms host RNA splicing via FAM50A to activate STAT3 and drive oncogenic cellular transformation
Source: mBio. 2025 Jun 12;16(7):e01293-25. doi: 10.1128/mbio.01293-25 (PMC12239556; doi:10.1128/mbio.01293-25)
Supplement: Supplemental Material — Tables S1 and S2; Figures S1 to S5. [file mbio.01293-25-s0001.pdf]

Supplemental Materials

KSHV Reprograms Host RNA Splicing via FAM50A to Activate STAT3 and Drive Oncogenic Cellular Transformation

Shenyu Sun, Ling Ding, Karla Paniagua, Xian Wang, Yufei Huang, Mario A Flores, Shou-Jiang Gao

Table S1 Summary of the clinical survival data of top nine splicing factors essential for KSHV-induced cellular transformation identified by Crispr-Cas9 screening in multiple cancer types

	NAA38	RBM22	U2AF2	PRMT5	MAGOH	FAM50A	RBMX2	SNRPB	PRPF40A	Case number
LIHC	0.00072	0.0031	0.0001	0.00022	0.083	0.026	0.0014	0.00013	0.079	365
ACC			0.056		0.00015	0.0014			0.0019	79
BLCA				0.028						406
COAD	0.067							0.021		279
ESCA						0.052	0.0043			184
HNSC				0.026			0.0036			519
KICH		0.013				0.016			0.084	65
KIRC						0.0047	0.057	0.004		531
KIRP		0.0071			0.0045		0.072	0.0001	0.012	287
LAML			0.012			0.0018		0.047		163
LGG			0.01		0.0001			0.0004	0.0019	511
LUAD									0.029	502
LUSC								0.086		494
MESO	0.028		0.0002		0.00016	0.0016		0.026	0.0056	85
PAAD									0.0003	177
SARC		0.056	0.012		0.011	0.072		0.083		259
THCA				0.078						504
UCEC							0.015		0.097	534
UVM			0.038			0.0019		0.013		80

P-values are based on comparison between the top 25% and the lower 75% cases.

Table S2 Primers and sequences

Primer name	Sequence
FAM50A_Rat_Forward	GAAGCAGAGGATTGCAGAGG
FAM50A_Rat_Reverse	TTTCTCGCTCCTTCACCAGT
Rat_SHP2_total_Forward	GGTTCCACGGTCACTTGTCT
Rat_SHP2_total_Reverse	TGGACTTGCTGTCATTGCTC
Rat_SHP2_long_Forward	ACAAGCTCTACTCCAGGGAAAC
Rat_SHP2_long_Reverse	CACCTTTCTCTCGGATGATG
Rat_SHP2_short_Forward	TCGGACAAGGAAACACAGAG
Rat_SHP2_short_Reverse	CGATGTCACAGTCCACACCT
Rat_SHP2_AS_validation_Forward	GCGCATGACTACACCTTACG
Rat_SHP2_AS_validation_Reverse	CCAGGTCCGAAAGTGGTACT
pCDH_Rat_SHP2_OE_Forward	TATGAATTCGCCACCATGGACTACAAAGACGATGACGACAAGATGACATCCCGGAGATGG
pCDH_Rat_SHP2_OE_Reverse	ATAGCGGCCGCTCATCTGAAACTCCTCTGCT
mRNA_LANA_Forward	GCAGACACTGAAACGCTGAA
mRNA_LANA_Reverse	AGGTGAGCCACCAGGACTTA
mRNA_vFLIP_Forward	GGATGCCCTAATGTCAATGC
mRNA_vFLIP_Reverse	GGCGATAGTGTTGGGAGTGT
mRNA_vCyclin_Forward	GCTGATAATAGAGGCGGGCAATGAG
mRNA_vCyclin_Reverse	GTTGGCGTGGCGAACAGAGAGGCAGTC
mRNA_RTA_Forward	CACAAAAATGGCGCAAGATGA
mRNA_RTA_Reverse	TGGTAGAGTTGGGCCTTCAGT T
mRNA_ORF57_Forward	AGGTCCCCCTCACCAGTAAA
mRNA_ORF57_Reverse	GAGGACGTGTGTTTTGACCG
mRNA_ORF65_Forward	ATATGTCGCAGGCCGAATAC
mRNA_ORF65_Reverse	CCACCCATCCTCCTCAGATA
mRNA_ORFK8_Forward	CATGCTGATGCGAATGTGC
mRNA_ORFK8_Reverse	AGCTTCAACATGGTGGGAGTG
mRNA_ORFK8.1_Forward	GTAACCGTGTGCCATTTTCTG
mRNA_ORFK8.1_Reverse	TCCCAGCAA TAA ACCCACAG
mRNA_ Rat_actin_Forward	GCAGGAGTACGATGAGTCCG
mRNA_ Rat_actin_Reverse	ACGCAGCTCAGTAACAGTCC
Genomic_ ORF71-73_Forward	ACTGAACACACGGACAACGG
Genomic_ORF71-73_Reverse	CAGGTTCTCCCATCGACGA
Genomic_ Rat_actin_Forward	ATGGATGACGATATCGCTGC
Genomic_ Rat_actin_Forward	CTTCTGACCCATACCCACCA

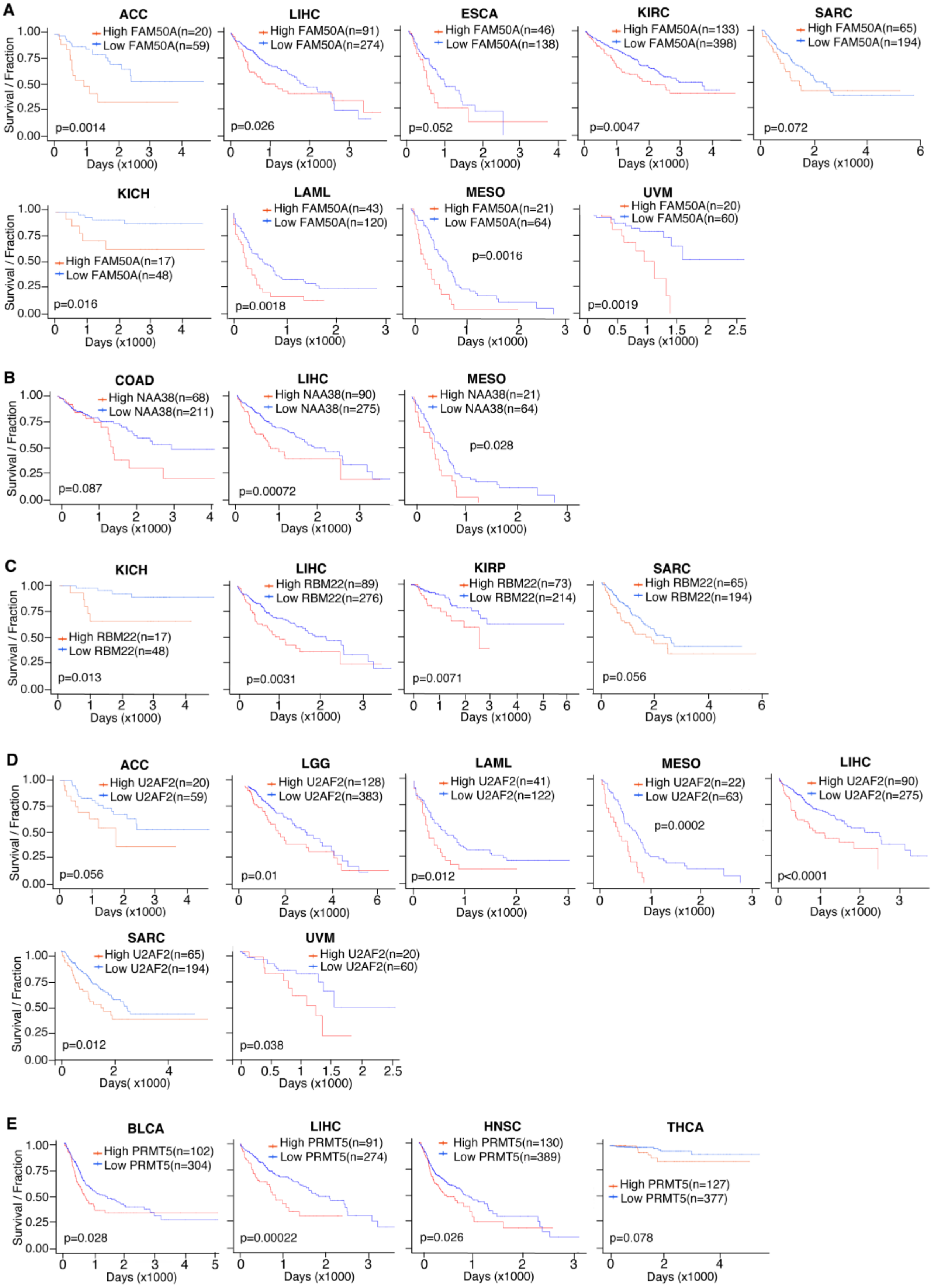
A
ACC
High FAM50A(n=20)
Low FAM50A(n=59)
p=0.0014
LIHC
High FAM50A(n=91)
Low FAM50A(n=274)
p=0.026
ESCA
High FAM50A(n=46)
Low FAM50A(n=138)
p=0.052
KIRC
High FAM50A(n=133)
Low FAM50A(n=398)
p=0.0047
SARC
High FAM50A(n=65)
Low FAM50A(n=194)
p=0.072
KICH
High FAM50A(n=17)
Low FAM50A(n=48)
p=0.016
LAML
High FAM50A(n=43)
Low FAM50A(n=120)
p=0.0018
MESO
High FAM50A(n=21)
Low FAM50A(n=64)
p=0.0016
UVM
High FAM50A(n=20)
Low FAM50A(n=60)
p=0.0019
B
COAD
High NAA38(n=68)
Low NAA38(n=211)
p=0.087
LIHC
High NAA38(n=90)
Low NAA38(n=275)
p=0.00072
MESO
High NAA38(n=21)
Low NAA38(n=64)
p=0.028
C
KICH
High RBM22(n=17)
Low RBM22(n=48)
p=0.013
LIHC
High RBM22(n=89)
Low RBM22(n=276)
p=0.0031
KIRP
High RBM22(n=73)
Low RBM22(n=214)
p=0.0071
SARC
High RBM22(n=65)
Low RBM22(n=194)
p=0.056
D
ACC
High U2AF2(n=20)
Low U2AF2(n=59)
p=0.056
LGG
High U2AF2(n=128)
Low U2AF2(n=383)
p=0.01
LAML
High U2AF2(n=41)
Low U2AF2(n=122)
p=0.012
MESO
High U2AF2(n=22)
Low U2AF2(n=63)
p=0.0002
LIHC
High U2AF2(n=90)
Low U2AF2(n=275)
p<0.0001
SARC
High U2AF2(n=65)
Low U2AF2(n=194)
p=0.012
UVM
High U2AF2(n=20)
Low U2AF2(n=60)
p=0.038
E
BLCA
High PRMT5(n=102)
Low PRMT5(n=304)
p=0.028
LIHC
High PRMT5(n=91)
Low PRMT5(n=274)
p=0.00022
HNSC
High PRMT5(n=130)
Low PRMT5(n=389)
p=0.026
THCA
High PRMT5(n=127)
Low PRMT5(n=377)
p=0.078
Survival / Fraction
Days (x1000)

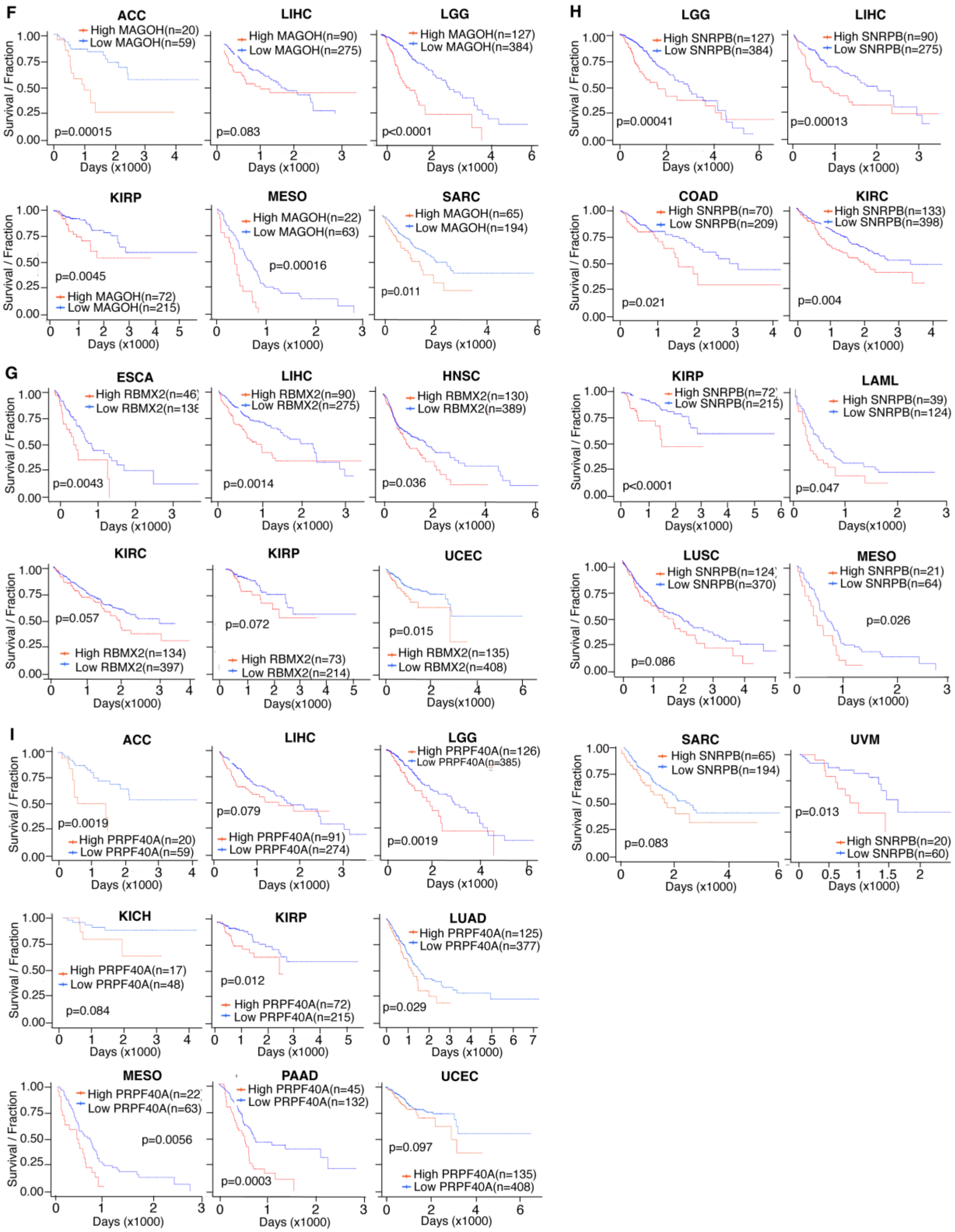
F
ACC
High MAGOH(n=20)
Low MAGOH(n=59)
p=0.00015
LIHC
High MAGOH(n=90)
Low MAGOH(n=275)
p=0.083
LGG
High MAGOH(n=127)
Low MAGOH(n=384)
p<0.0001
KIRP
p=0.0045
High MAGOH(n=72)
Low MAGOH(n=215)
MESO
High MAGOH(n=22)
Low MAGOH(n=63)
p=0.00016
SARC
High MAGOH(n=65)
Low MAGOH(n=194)
p=0.011
G
ESCA
High RBMX2(n=46)
p=0.0043
LIHC
High RBMX2(n=90)
Low RBMX2(n=275)
p=0.0014
HNSC
High RBMX2(n=130)
Low RBMX2(n=389)
p=0.036
KIRC
p=0.057
High RBMX2(n=134)
Low RBMX2(n=397)
KIRP
p=0.072
High RBMX2(n=73)
Low RBMX2(n=214)
UCEC
p=0.015
High RBMX2(n=135)
Low RBMX2(n=408)
I
ACC
p=0.0019
High PRPF40A(n=20)
Low PRPF40A(n=59)
LIHC
p=0.079
High PRPF40A(n=91)
Low PRPF40A(n=274)
LGG
High PRPF40A(n=126)
Low PRPF40A(n=385)
p=0.0019
KICH
High PRPF40A(n=17)
Low PRPF40A(n=48)
p=0.084
KIRP
p=0.012
High PRPF40A(n=72)
Low PRPF40A(n=215)
LUAD
High PRPF40A(n=125)
Low PRPF40A(n=377)
p=0.029
MESO
Low PRPF40A(n=63)
p=0.0056
PAAD
High PRPF40A(n=45)
Low PRPF40A(n=132)
p=0.0003
UCEC
p=0.097
High PRPF40A(n=135)
Low PRPF40A(n=408)
H
LGG
High SNRPB(n=127)
Low SNRPB(n=384)
p=0.00041
LIHC
High SNRPB(n=90)
Low SNRPB(n=275)
p=0.00013
COAD
High SNRPB(n=70)
Low SNRPB(n=209)
p=0.021
KIRC
High SNRPB(n=133)
Low SNRPB(n=398)
p=0.004
KIRP
Low SNRPB(n=215)
p<0.0001
LAML
High SNRPB(n=39)
Low SNRPB(n=124)
p=0.047
LUSC
Low SNRPB(n=370)
p=0.086
MESO
High SNRPB(n=21)
Low SNRPB(n=64)
p=0.026
SARC
High SNRPB(n=65)
Low SNRPB(n=194)
p=0.083
UVM
p=0.013
High SNRPB(n=20)
Low SNRPB(n=60)
Survival / Fraction
Days (x1000)

FIG S1 Clinical survival data of top nine splicing factors essential for KSHV-induced cellular transformation identified by CRISPR-Cas9 screening in multiple cancer types. (A-I) Kaplan-Meier survival curves for the top nine splicing factors in various cancers, including FAM50A (A), NAA38 (B), RBM22 (C), U2AF2 (D), PRMT5 (E), MAGOH (F), RBMX2 (G), SNRPB (H), and PRPF40A (I). Survival outcomes were analyzed based on gene expression levels, with statistical significance determined by log-rank tests.

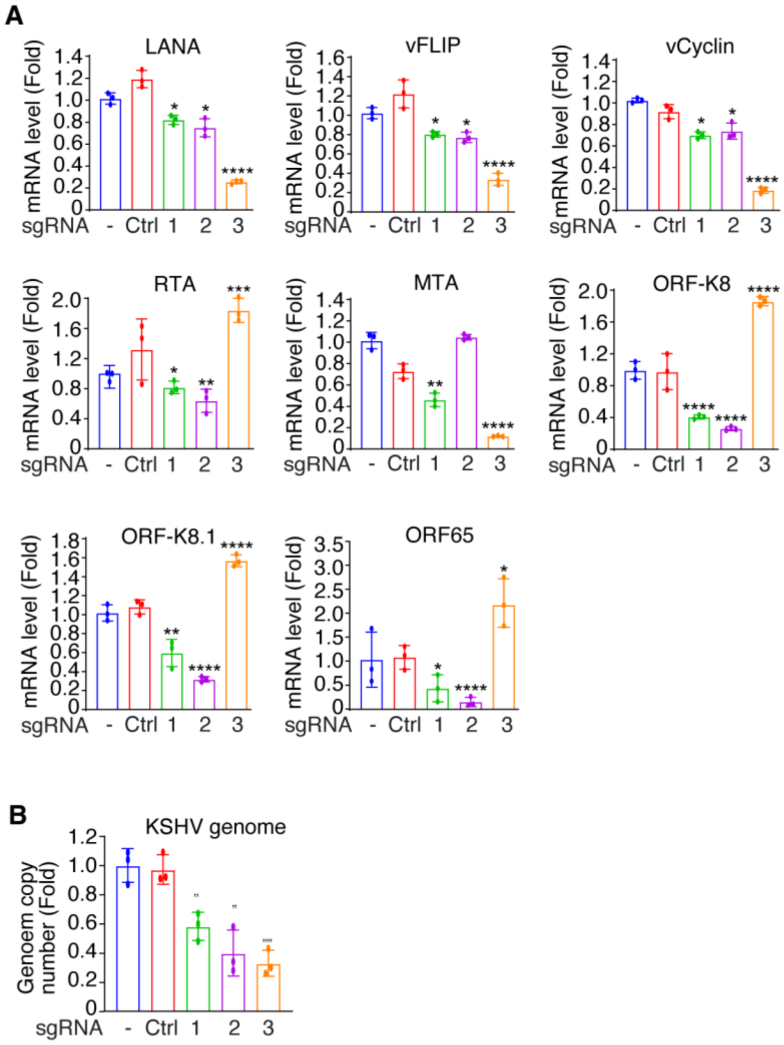

FIG S2 The effect of FAM50A knockout on viral gene expression and viral genome copy number per cell in KMM cells. (A) RT-qPCR examination of the expression of viral genes LANA, vFLIP, vCyclin, RTA, MTA, ORF-K8, ORF-K8.1 and ORF65 following FAM50A knockout in KMM cells. (B) qPCR examination of viral genome copy number per cell following FAM50A knockout in KMM cells. Data are presented as mean ± 95% CI and *P*-values (**P* < 0.05; ***P* < 0.01, ****P* < 0.001, *****P* < 0.0001) were determined using Student's *t*-test.

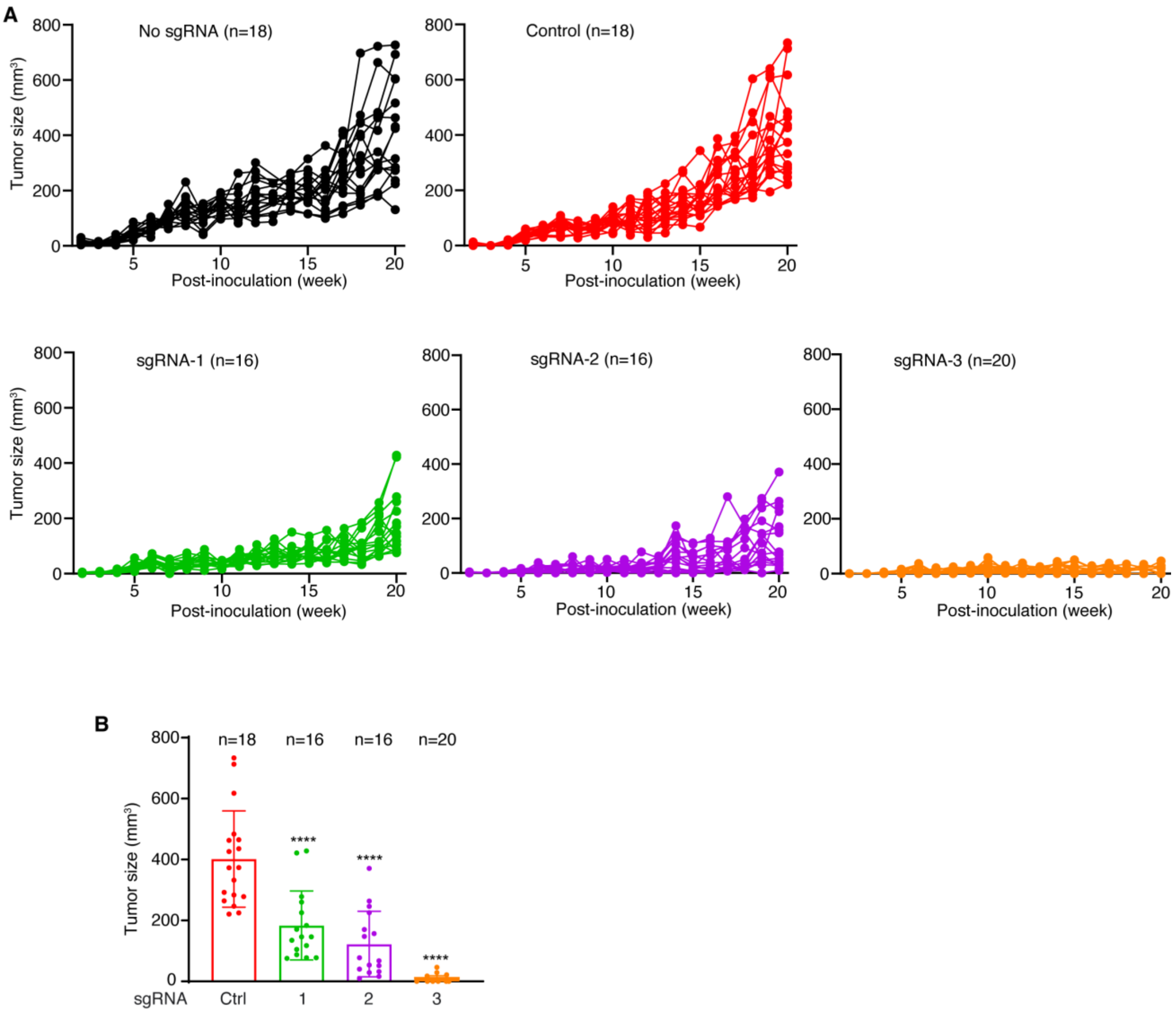

FIG S3 (A) Tumor growth trajectories of individual tumors in nude mice implanted with FAM50A knockout KMM cells (sgFAM50A-1, 2 and 3), no sgRNA KMM-Cas9 cells, and control KMM-Cas9 cells with scrambled sgRNAs. (B) Tumor volumes at the study endpoint for each group. Statistical comparisons were performed between each sgRNA-treated group and the Control. Data are presented as mean ± 95% confidence interval (CI), with significance determined using Student's *t*-test. (****$P < 0.0001$).

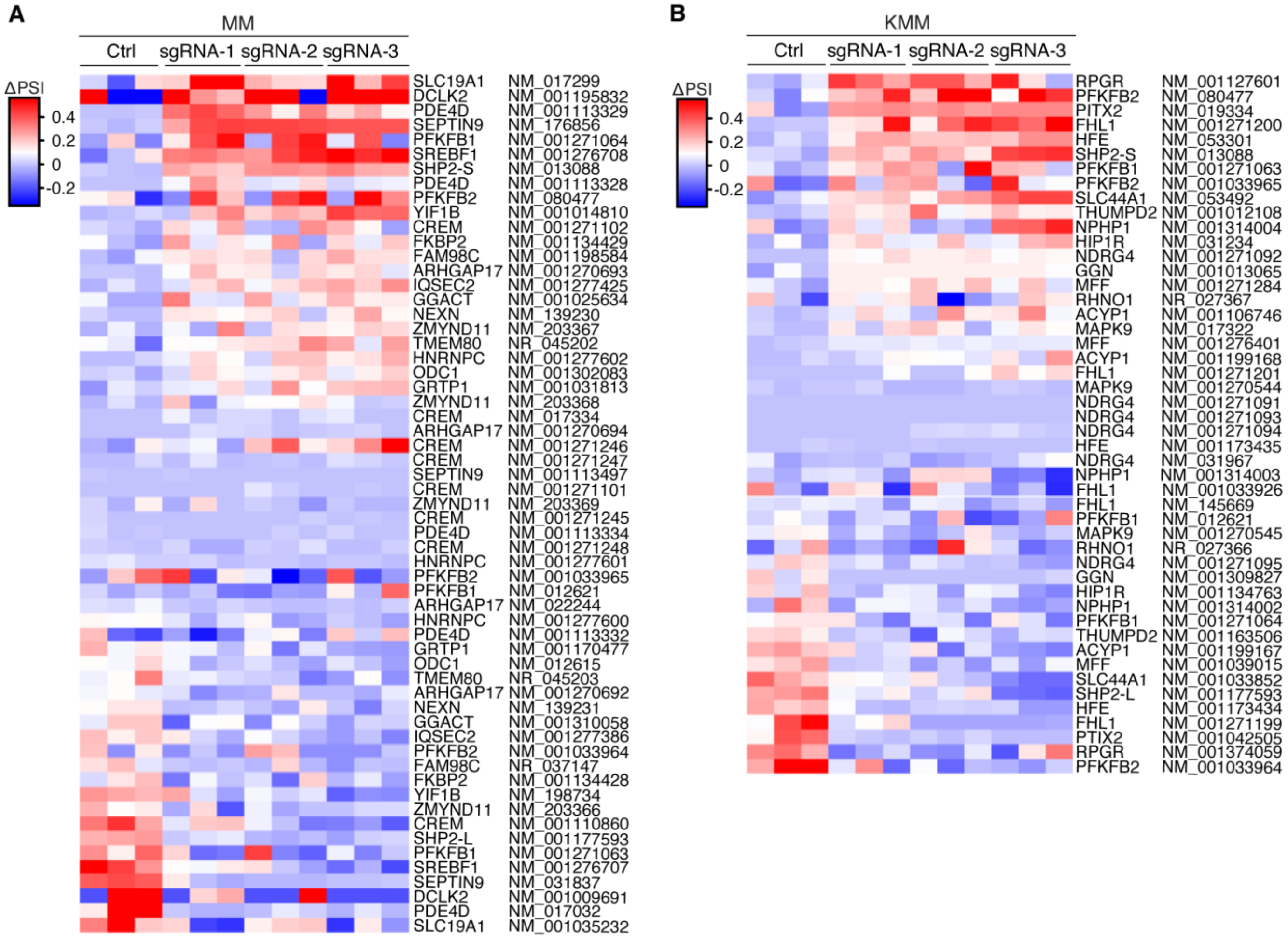

FIG S4 Alternative splicing analysis in FAM50A knockout cells. (A-B) Heatmaps displaying ΔPSI values for all differential spliced transcripts between MM *vs.* FAM50A knockout MM cells (A), and KMM *vs.* FAM50A knockout KMM cells (B). ΔPSI values were calculated based on three independent biological replicates per group. Differential spliced transcripts are defined as those that have significant percentage changes among all the spliced transcripts of the gene.

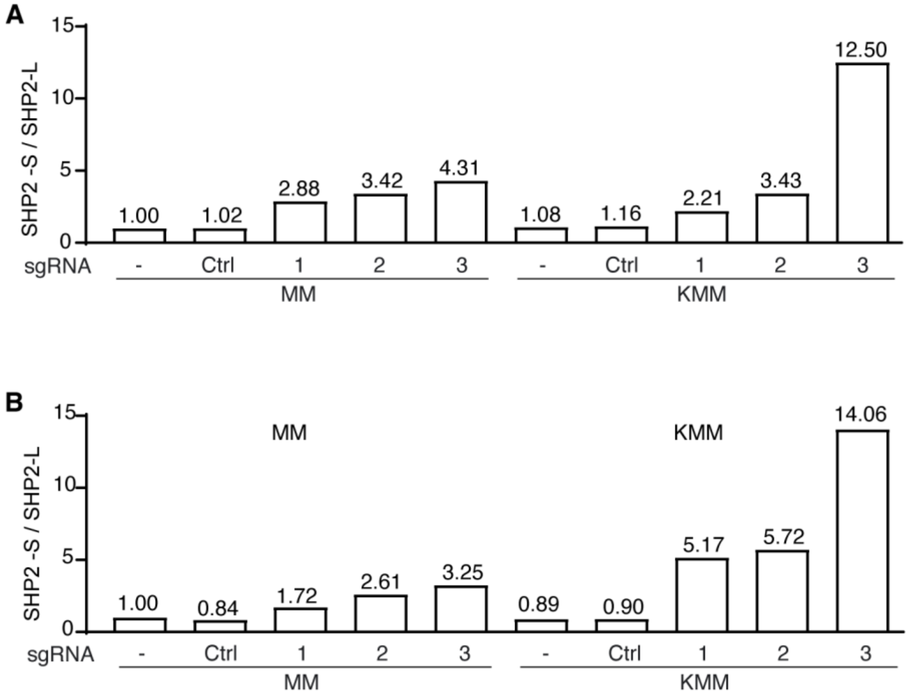

FIG S5 Quantification of SHP2 isoform ratios. (A) SHP2-S/SHP2-L ratio derived from the data in Figure 6A. (B) SHP2-S/SHP2-L ratio calculated from the data in Figure 6C.
